# Impact of HDAC Inhibitors on Protein Quality Control Systems: Consequences for Precision Medicine in Malignant Disease

**DOI:** 10.3389/fcell.2020.00425

**Published:** 2020-06-03

**Authors:** Linda Anna Michelle Kulka, Pia-Victoria Fangmann, Diana Panfilova, Heidi Olzscha

**Affiliations:** Medical Faculty, Institute of Physiological Chemistry, Martin-Luther-University Halle-Wittenberg, Halle (Saale), Germany

**Keywords:** autophagy, bromodomain-containing protein, epigenetic drug, histone deacetylase inhibitor, molecular chaperone, precision medicine, protein quality control, ubiquitin proteasome system

## Abstract

Lysine acetylation is one of the major posttranslational modifications (PTM) in human cells and thus needs to be tightly regulated by the writers of this process, the histone acetyl transferases (HAT), and the erasers, the histone deacetylases (HDAC). Acetylation plays a crucial role in cell signaling, cell cycle control and in epigenetic regulation of gene expression. Bromodomain (BRD)-containing proteins are readers of the acetylation mark, enabling them to transduce the modification signal. HDAC inhibitors (HDACi) have been proven to be efficient in hematologic malignancies with four of them being approved by the FDA. However, the mechanisms by which HDACi exert their cytotoxicity are only partly resolved. It is likely that HDACi alter the acetylation pattern of cytoplasmic proteins, contributing to their anti-cancer potential. Recently, it has been demonstrated that various protein quality control (PQC) systems are involved in recognizing the altered acetylation pattern upon HDACi treatment. In particular, molecular chaperones, the ubiquitin proteasome system (UPS) and autophagy are able to sense the structurally changed proteins, providing additional targets. Recent clinical studies of novel HDACi have proven that proteins of the UPS may serve as biomarkers for stratifying patient groups under HDACi regimes. In addition, members of the PQC systems have been shown to modify the epigenetic readout of HDACi treated cells and alter proteostasis in the nucleus, thus contributing to changing gene expression profiles. Bromodomain (BRD)-containing proteins seem to play a potent role in transducing the signaling process initiating apoptosis, and many clinical trials are under way to test BRD inhibitors. Finally, it has been demonstrated that HDACi treatment leads to protein misfolding and aggregation, which may explain the effect of panobinostat, the latest FDA approved HDACi, in combination with the proteasome inhibitor bortezomib in multiple myeloma. Therefore, proteins of these PQC systems provide valuable targets for precision medicine in cancer. In this review, we give an overview of the impact of HDACi treatment on PQC systems and their implications for malignant disease. We exemplify the development of novel HDACi and how affected proteins belonging to PQC can be used to determine molecular signatures and utilized in precision medicine.

## Introduction

### Lysine Acetylation and Histones

**FIGURE 1:**
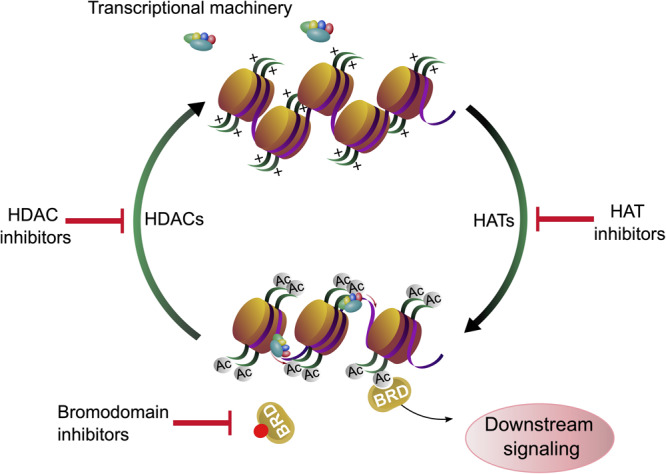
Histone acetylation, deacetylation and chromatin accessibility. Gene expression is regulated by lysine acetylation of histone proteins. Histone acetyl transferases (HATs) catalyze the transfer of acetyl groups onto proteins. Acetylation of histones affects the chromatin structure and can facilitate gene expression. Histone deacetylases (HDACs) remove the acetylation marks from histones. Acetylation and deacetylation can be modulated by histone acetyl transferase inhibitors (HATi) and histone deacetylase inhibitors (HDACi), respectively. The acetylation marks are recognized by bromodomain containing proteins (BRD) whose binding can be blocked by bromodomain inhibitors (BDi).

Lysine acetylation at the ε-amino group is one of the most abundant posttranslational modifications (PTM) in eukaryotic cells. Due to the neutralization of positive charge on lysine residues, acetylated proteins can interact with different molecules and adopt different folds. Thus, reversible lysine acetylation plays a crucial role in many biological processes, including gene expression, chromatin remodeling, cell cycle control, cell signaling and protein quality control (PQC) (Kouzarides, 2000). Protein acetylation is a classic example of a reversible PTM which can accommodate the needs of cells and reflect responses to environmental changes. Traditionally, enzymes which “write” the acetylation mark on lysine residues are termed histone acetyltransferases (HAT), enzymes which remove the acetylation mark from lysine residues in proteins, the erasers, are named histone deacetylases (HDAC) (New et al., 2012). The name “histone” deacetylases reflects the fact that many lysine residues in histones are acetylated and explains to some extent the epigenetic aspect of histone deacetylases and therefore histone deacetylase inhibitors (HDACi). However, it has been demonstrated that thousands of other proteins can be acetylated and deacetylated both in the cytoplasm and in the nucleus (Choudhary et al., 2009). Consequently, most HDACs act on proteins which occur in the nucleus and in the cytoplasm, implying that HDACi can affect not only histones, but all other proteins, depending on their class specificity. Bromodomain (BRD)-containing proteins act as “readers” and recognize acetylation marks. They translate acetylation signals to downstream signaling cascades, leading for example to further histone modifications or chromatin remodeling, finally shaping the cell into diverse phenotypes (Filippakopoulos and Knapp, 2014; Figure 1).

Histones are proteins, which organize the DNA into a compact form called nucleosome (Olzscha et al., 2015), and the bond strength between histone units and DNA can be determined by the acetylation of lysine residues of histones (Luger and Richmond, 1998). The acetylation of distinct lysine residues of histones (H2A, H2AX, H2B, H3, and H4) has different functions. Generally, histone acetylation is associated with transcriptional activation: If the histones are acetylated at many lysine residues, the nucleosome is present in its open form and genes can be transcribed by RNA-polymerases. It is assumed that the acetylation neutralizes the positive charge of the amino terminus and therefore the binding between histones and DNA is weakened (Annunziato and Hansen, 2018). Consequently, lysine residues regain their positive charge upon deacetylation and the affinity of the negatively charged DNA phosphate backbone to the amino terminus of histones is increased (Shahbazian and Grunstein, 2007). Other functions of lysine acetylation are DNA repair (H2AX on Lys5 and Lys36, H2A on Lys5, H3 on Lys9, 14, 18, 23, 27, 36, 56, and H4 on Lys5, 8, 12, 16, 91), histone deposition (H3 on Lys9,14 and H4 on Lys5, 12), transcriptional elongation (H3 on Lys14 and H4 on Lys8), chromatin assembly (H3 on Lys56), telomeric silencing (H4 on Lys12), chromatin decondensation (H4 on Lys16) and DNA replication (H4 on Lys91) (Koprinarova et al., 2016). Acetylation of H2A and H2B are mostly taking part in transcriptional activation, while acetylation on H2AX, H3 and H4 lysine residues can have different effects. In conclusion, deacetylation of histone proteins with HDACs is required for chromatin remodeling, many downstream processes and regulatory pathways (Wade, 2001; Figure 1).

### HDAC Classification and Characterization

Eighteen human HDACs have been described and classified into four groups. We provide only a short introduction about the different HDAC classes, as there are many comprehensive reviews which give overviews about the HDAC classes and also chemical classes of HDACi, for example (New et al., 2012) or (Seto and Yoshida, 2014). The classification in *Homo sapiens* is based on the HDAC’s homology to yeast proteins (Dokmanovic et al., 2007). HDAC1, 2, 3, and 8 belonging to class I are homolog to the yeast RPD3 protein and are localized in the nucleus; they are involved in cell survival and proliferation. The class II HDACs (HDAC4, 5, 6, 7, 9, and 10) are supposed to play a tissue-specific role (Lagger et al., 2002). They are homolog to the yeast HDAC HDA1 (histone deacetylase 1) and can be found in the nucleus or cytoplasm. HDAC4, 5, 7, and 9 belong to class IIa and contain only one catalytic domain, while class IIb HDACs (6 and 10) have two catalytic domains and can only be detected in the cytoplasm. HDACs of class I and II contain Zn^2+^ in their catalytic sites, and thus are known as Zn^2+^-dependent HDACs. The HDACs from class III (SIRT1-7) are homolog to the Sir2 yeast protein. They do not contain Zn^2+^ in their catalytic sites, but require NAD^+^ for their enzymatic activity (Bolden et al., 2006). Class IV consists of only one protein, HDAC11. Regions in its catalytic center are similar to both class I and II sequences; hence, it is also classified as Zn^2+^-dependent HDAC (Gao et al., 2002).

The abundance and enzymatic activity of HDACs in cells is regulated on various levels e.g., by changes in gene expression, protein complex formation, PTMs, subcellular localization and by the availability of metabolic cofactors (Sengupta and Seto, 2004).

### HDAC Inhibitors (HDACi)

Histone deacetylase inhibitors suppress HDAC activity. There are six structurally defined classes of HDACi: small molecular weight carboxylates, hydroxamic acids, benzamides, epoxyketones, cyclic peptides and hybrid molecules. They mainly act on HDACs of the classes I, II and IV by binding the Zn^2+^-containing catalytic domain (Drummond et al., 2005). The first discovered HDACi, the natural antifungal antibiotic trichostatin A (TSA), belongs to hydroxamic acid-type chelators (Yoshida et al., 1990), and the TSA structural analog vorinostat, also known as suberoylanilide hydroxamic acid (SAHA) was the first HDACi being approved by the U.S. Food and Drug Administration (FDA). The other three HDACi approved by the FDA so far are romidepsin, belinostat and panobinostat (Yoon and Eom, 2016). NAD^+^-dependent class III HDACs are inhibited by NAD^+^ and its derivates, dehydrocoumarin, splitomycin, 2-OH-naphtaldehyde, sirtinol and M15 (Porcu and Chiarugi, 2005). However, in this review, we focus on the “classic” HDACs belonging to the classes I, II and IV and their respective HDACi.

Vorinostat (Zolinza^®^) was approved in October 2006 for treatment of advanced primary cutaneous T-cell lymphoma (CTCL) (Mann et al., 2007). Romidepsin (Istodax^®^) was licensed for CTCL treatment in 2009 (Whittaker et al., 2010), and later, in 2011 for peripheral T-cell lymphoma (PTCL) (Coiffier et al., 2012). Belinostat (Beleodaq^®^) was approved by the FDA in 2014 for the treatment of PTCL. The fourth approved HDACi panobinostat (Farydak^®^) was licensed in 2015 for the treatment of multiple myeloma (MM).

As already mentioned, HDACi have a profound effect on the structure of chromatin and therefore on the transcriptional activity of the affected gene chromatin regions. This is why HDACi can be seen as established epigenetic modulators, since they affect the read-out of genes without changing the DNA sequence (Olzscha et al., 2015).

### Epigenetics and Cancer

Epigenetics can be defined as inherited changes in phenotypes or entities, which are not encoded in the nucleotide sequence of the organism, but are passed on to daughter cells (Olzscha et al., 2015). Exogenous influences and altered environmental conditions can change epigenetic signatures and may give a hint about the origin of different malignancies, such as cancer or neurological disorders (Tsankova et al., 2007; John and Rougeulle, 2018). One appearance of epigenetics can be biochemical post-replicative modifications of the DNA-sequence, either through alteration of single bases or as described above in proteins (Handy et al., 2011).

Traditionally, cancer has been defined as a group of diseases leading to uncontrolled cell proliferation caused by genetic mutations in tumor-suppressor genes and oncogenes or chromosomal abnormalities (Hanahan and Weinberg, 2000). However, cancer may also be driven by epigenetic changes (Baylin and Ohm, 2006). According to its definition, epigenetic changes can be heritable and also known as epimutations, equivalent to mutations; however, some changes, in particular, histone deacetylation that repress gene expression by wrapping DNA more tightly, are not heritable, but have been also described as “epigenetic” (Berger et al., 2009). Thus, acetylation can influence transcriptional regulation, cell cycle control, apoptosis and autophagy, but also the activity of further proteins that maintain protein homeostasis, which will be described below (Nihira et al., 2017).

### Objectives of the Review

Since thousands of proteins can be acetylated by HATs and deacetylated by HDACs, HDACi will not only act on an epigenetic level, but will also influence crucial protein functions, especially in PQC systems. These systems and the underlying effects will be described in this review and how this knowledge is utilized to develop combination therapies of HDACi and modulators of PQC processes. Clinical trials with HDACi alone or in combination are systematically evaluated for their potential to identify novel targets of PQC systems and their effect on epigenetic modulation. We also exemplify the development of novel HDACi which are in clinical trials, provide evidence that PQC systems are involved and how the underlying proteins can be used as biomarkers. Finally, we give an outlook on current and future HDACi development, its impact on proteostasis and how this knowledge can be utilized to improve precision medicine for cancer patients.

## HDACs in Epigenetics and Protein Quality Control Systems

### The Role of HDACs in Epigenetics and Cancer Cells

Recent studies suggest that cancer cells have increased concentrations of HDACs. For instance, according to clinical and preclinical studies, class I HDACs may stimulate cell proliferation and survival (Yoon and Eom, 2016). It has been shown that HDAC1 is overexpressed in prostate (Halkidou et al., 2004), gastric (Choi et al., 2001), colon (Wilson et al., 2006), and breast (Zhang et al., 2005) carcinomas. HDAC2 is reported to be responsible for the loss of adenomatous polyposis coli (APC) expression in colorectal cancer (CRC) (Zhu et al., 2004) and displays increased expression in cervical (Huang et al., 2005) and gastric (Song et al., 2005) carcinomas. HDAC3 and HDAC6 are also reported to show increased concentration in colon and breast carcinoma cells (Zhang et al., 2004; Wilson et al., 2006).

One of the problems in cancer is the heterogeneity which can occur from different mutations and/or different epigenetic patterns. Genomic variability occurs sometimes even if the cells display similar phenotypes or when there are differences in the phenotype, even though the cells originate from one tumor population (Cantor and Sabatini, 2012). These observations strengthen the hypothesis that epigenetics plays an important role in cancer development. Tumor suppressor genes might be silenced and oncogenes activated upon epigenetic changes without any influence on the genotype (Llinàs-Arias and Esteller, 2017). It is hypothesized that molecular chaperones such as the heat shock protein 90 (HSP90) act as regulators of the genotype-to-phenotype interplay and offer an evolutionary buffer to protect cells from malignant transformation (Whitesell and Lindquist, 2005; Jarosz, 2016).

The first discovered non-histone target of HATs and HDACs was p53 (Sakaguchi et al., 1998; Liu et al., 1999), a tumor suppressor protein that is able to bind DNA. Therefore, it can affect chromatin structure and can epigenetically change gene expression, whereby the binding is regulated by acetylation. Furthermore, acetylated p53 is able to induce apoptosis and autophagy (Fridman and Lowe, 2003; Mrakovcic and Fröhlich, 2018). Apoptosis is a form of highly controlled, energy-dependent programmed cell death, and malignant cells often depend on inherent or acquired mechanisms to resist cell death, which is seen as a hallmark of cancer (Hanahan and Weinberg, 2000). It is still under investigation, whether acetylation alters the interaction with other proteins or whether it results in a conformational change of p53 (Mrakovcic et al., 2018).

Another example for a silenced tumor suppressor in cancer cells is p21, which acts as a cyclin-dependent kinase (CDK) inhibitor. As p21 plays a crucial role in the regulation of CDKs, its expression has a big impact on cancer growth. Silencing of p21 occurs as a result of hypoacetylation of its promotor and consequently, it has been shown that HDAC1 inhibits the promotor by binding at the SP1-site (specificity protein 1) and competes with p53 activating the promoter of p21 (Gui et al., 2004). Using HDACi, there are two different mechanisms known leading to an enhanced expression of p21, with one of them p53-independent and the other p53-dependent. In the p53-independent mechanism, treatment with HDACi results in a release of HDAC1. Therefore, the promotor loses its repression and the gene of p21 is transcribed. The p53-dependent mechanism displays an enhanced expression of p21, as the HDACi induces acetylation of p53 resulting in a higher binding affinity to the p21 promotor (Ocker and Schneider-Stock, 2007).

HDAC1 is also known to bind ETO (eight-twenty-one), which can be fused with AML1 (acute myeloid leukemia 1). This fusion protein AML1-ETO arises as a result of a *t*(8;21) translocation and it has been shown that HDACi is efficient as a treatment against AML, suggesting a non-epigenetic effect. A study using valproic acid (VPA) as an HDACi reported a dissociation of the AML1-ETO/HDAC1 complex from the AML1-ETO promotor. Other studies reported a proteasomal degradation of AML-ETO after HDACi treatment, again demonstrating the importance of the PQC systems in cancer cells (Hug and Lazar, 2004).

### The Role of Protein Quality Control Systems in Cancer Cells

**FIGURE 2:**
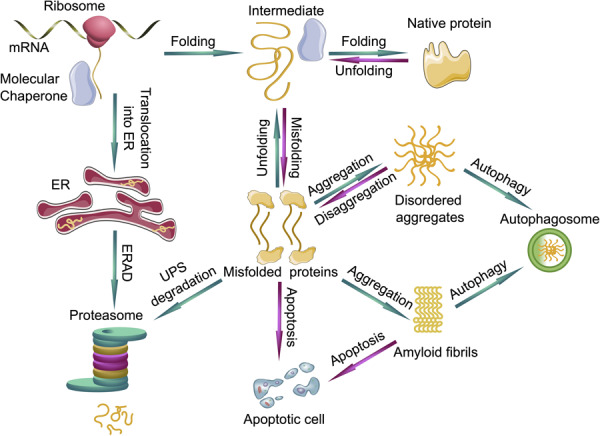
Protein quality control systems and their impact on protein folding, misfolding and aggregation. Molecular chaperones assist in the folding of nascent and unfolded proteins. If the folding fails, the unfolded or misfolded proteins are able to form disordered aggregates or even highly ordered amyloid fibrils. The ubiquitin proteasome system (UPS) can degrade the prefibrillar misfolded proteins from the cytoplasm and misfolded proteins from the endoplasmic reticulum (ER) *via* endoplasmic reticulum-associated protein degradation (ERAD), whereas larger aggregates can be degraded by autophagy. If protein quality control systems fail, cells can undergo apoptosis. Green arrows represent functioning PQC systems, eliminating cytotoxic species from cells and are pro-survival, whereas magenta arrows represent deleterious events where PQC systems fail, leading to cell death.

In order to ensure the correct protein folding and protection of the proteome, eukaryotic cells developed a complex PQC system. Molecular chaperones, the ubiquitin proteasome system (UPS) and autophagy form a complex network which maintains the integrity of the proteome (Mogk and Bukau, 2006; Chen et al., 2011; Olzscha, 2019; Figure 2).

#### Molecular Chaperones

Most molecular chaperones are heat shock proteins and *vice versa*, they can be ATP-dependent and exert different mechanisms of assistance in protein folding. They are classified due to their sequence homology to specific heat shock proteins and their molecular mass: The HSP100/Clp-family, HSP90-family, HSP70-family, HSP60/GroEL-family and small heat shock proteins (sHSPs) (Jeng et al., 2015). All of them are known to assist proteins to fold correctly, especially complex proteins. They interact specifically with proteins and accelerate the folding, without being part of their final structure (Hartl and Hayer-Hartl, 2009). PTMs can alter protein folding (Santos and Lindner, 2017; Olzscha, 2019), and molecular chaperones are also known to be regulated by PTMs, including acetylation. For instance, it has been demonstrated that romidepsin stabilizes the acetylation of HSP70 leading to an increased binding of oncogenic proteins, which are normally stabilized by HSP90 (Cloutier and Coulombe, 2013). HSP90 is a ubiquitous occurring molecular chaperone, which supports a variety of proteins in their folding process. Accordingly, it affects many cellular processes, such as cell proliferation and signal transduction and plays a crucial role in cancer development (Scroggins et al., 2007).

Transformed cells can adapt metabolites for tumorigenesis, which can lead to further epigenetic modifications and subsequently tumor progression (Pavlova and Thompson, 2016). On the one hand, HSP90 can control this metabolic rewiring (Condelli et al., 2019), on the other hand, it determines the transcription of specific oncogenes. HSP90 can bind the chromatin directly or control the transcription factors of the genes (Khurana and Bhattacharyya, 2015). Thus, HSP90 can be seen as a paradigm for the interplay between molecular chaperones and epigenetics. If HSP90 is hyperacetylated either by knock-down of HDAC6 or by administration of an HDACi, its activity is impaired (Kovacs et al., 2005). This has been demonstrated with the treatment of the pan-HDACi panobinostat (LBH589), an anti-cancer drug approved by the FDA against MM (Yang et al., 2008; FDA, 2015).

Another example is given by the molecular chaperone HSP70, which supports the folding and refolding of proteins and can prevent aggregation or even refold aggregated proteins to a certain extent (Mayer and Bukau, 2005). It has been reported that its promotor is hypermethylated in cancer cells and the expression of HSP70 is enhanced by histone methylation. In a human oral squamous cell carcinoma cell line, the methylation of histone H3 at the lysine residues Lys4 and Lys9 enhanced the expression of HSP70 (Ban et al., 2019).

#### The Ubiquitin Proteasome System (UPS)

The UPS degrades proteins into oligopeptides (Ravid and Hochstrasser, 2008) and since ubiquitin is attached to lysine residues, it implies that competition with acetylation is generally possible (Caron et al., 2005). The proteasome recognizes the polyubiquitin chain, unfolds the target protein and finally degrades it. Target proteins can be metabolic enzymes, transcription factors and cell cycle regulating proteins, including cyclins and CDK-inhibitors (Schrader et al., 2009). All of these proteins are known to play a crucial role in cancer, for instance, metabolic enzymes are important for maintaining the tumor microenvironment and nutrient availability. In cancer cells, their protein level and occurrence can be altered as a result of mutations and non-genetic changes, including the adaption of metabolic enzymes, which are normally degraded by the UPS (Wegiel et al., 2018). Especially glycolysis and the tricarboxylic acid cycle are well analyzed targets (Yu et al., 2017). As cancer cells have a high proliferation rate, they need high amounts of ATP as energy supply and nutrients, including lipids, nucleotides and amino acids. This higher proliferation rate also led to changes in cell cycle, affecting regulatory proteins such as cyclin and CDK-inhibitors (Deshpande et al., 2005).

Perhaps the best-known protein associated with cancer is the before-mentioned tumor suppression protein p53, which is inactivated in many types of cancer. The concentration of p53 is regulated by polyubiquitination and subsequent proteasomal degradation, in particular by mouse double minute 2 homolog (MDM2) (Patel and Player, 2008), which is a RING (really interesting new gene) E3-ligase. It can form a complex with p300/CBP (CBP, CREB binding protein; CREB, cAMP response-element binding protein) resulting in the polyubiquitination and degradation of p53 (Grossman et al., 1998). Inhibition of the proteasome can prevent this degradation (Harris et al., 2008); however, the p53 gene is often mutated in cancer cells, leading to the conclusion that intervention on the transcriptional level seems to be more promising. However, proteasome inhibitors (PI) were tested together with HDACi and synergistic effects of PIs with HDACi were proven, e.g., the FDA-approved inhibitor bortezomib with the pan-HDACi vorinostat (Johnson, 2015). Thus, the UPS plays a relevant role with regards to the treatment of cancer, and examples of clinical trials in this combination are given in section “HDAC Inhibitors and Proteasome Inhibitors” of this review.

Furthermore, proteasomes degrade proteins that are recognized as misfolded, a process which needs to be distinguished from protein degradation being a regulatory step to control the half-life of a protein (Figure 2). Protein misfolding can occur spontaneously within the cell, or the protein failed to fold correctly after its biogenesis. In case molecular chaperones are unable to assist in protein folding, the misfolded proteins are recognized by specific adapter proteins such as molecular chaperones together with the carboxy terminus of heat shock protein 70-interacting protein (CHIP) and marked for degradation by the UPS (Meccariello et al., 2014). PTMs can be one reason for protein misfolding and in some cases result in aberrant degradation of these proteins *via* the UPS (Olzscha, 2019).

Glucose-regulated protein 78 (GRP78) is another HSP that plays a crucial role in regulating the unfolded protein response (UPR). This pathway is induced by ER (endoplasmic reticulum) stress. For instance, an increase of accumulated misfolded protein in the ER lumen can lead to ER stress and associated pathways. There are also molecular chaperones present in the ER to prevent misfolding and aggregation of proteins, but they can also fulfill special tasks within the ER. One of the molecular chaperones is binding immunoglobulin protein (BiP), also known as GRP78 (78-kDa glucose-regulated protein). BiP can recognize and bind misfolded proteins in the ER, leading to a dissociation and activation of the protein kinase RNA-like endoplasmic reticulum kinase (PERK), activating transcription factor 6 (ATF6) and inositol-requiring enzyme 1 (IRE1) (Warri et al., 2018). ATF6 can induce the Akt/mTOR (mammalian target of rapamycin) pathway and promotes the transcription of different genes, including the autophagy-related genes 12 (ATG12) and 5 (ATG5) regulating autophagy (Yan et al., 2015). Autophagy is another PQC system, which is described below. It is known that different HDACi, for example vorinostat (Kahali et al., 2010), YCW1 and OSU-HDAC2, can induce ER stress causing autophagy (see also section “HDAC Inhibitors Affecting Protein Quality Control Systems”).

#### Autophagy

Autophagy is another part of the PQC systems, which allows cells to degrade cytoplasmic constituents and to remove unnecessary or dysfunctional proteins (Chun and Kim, 2018). Misfolded and aggregated proteins can be degraded by autophagy, especially, if molecular chaperones or the UPS are not able to cope with the amount of misfolded proteins. These aggregated proteins are capable to form amyloid structures which are the underlying cause for proteinopathies such as Alzheimer’s disease (AD) or amyotrophic lateral sclerosis (ALS). In superoxide dismutase 1 (SOD1) mice, the impairment of the UPR and autophagy was proven to be partly responsible for the pathophysiology of ALS (Ruegsegger and Saxena, 2016).

One can differentiate between macro-, micro- and chaperone-mediated autophagy (CMA) (Glick et al., 2010). Macroautophagy is the main pathway in the cell to degrade damaged cell organelles or aggregated proteins. In the process of engulfment, an autophagosome is built, which is a circular double-membrane structure that encloses the target protein. The autophagosome comes in close proximity to the lysosome and fuses with it, forming the autolysosome, where proteins get hydrolyzed *via* lysosomal hydrolases in an acidic environment (Feng et al., 2014). During microautophagy, proteins are also degraded *via* acidic lysosomal hydrolases; however, they are directly engulfed by vesicles originating from lysosomes (Li et al., 2012). During CMA, HSP70 chaperones recognize proteins containing a KFERQ-like motif. This leads to the formation of a CMA-substrate/chaperone complex, which is located to the lysosomal receptor LAMP-2A (lysosome-associated membrane protein). The protein is unfolded and translocated across the lysosomal membrane where it is degraded (Cuervo and Wong, 2014).

Autophagy is regulated by autophagy-related genes (ATG) (Wesselborg and Stork, 2015). In cancer cells, autophagy can be disturbed in a way that either they degrade apoptotic mediators, which would normally kill the cancer cells, or the survival of starving cancer cells is prolonged (Mathew et al., 2007; Fernald and Kurokawa, 2013). However, autophagy acts as a tumor suppressor in non-cancerous cells (Kung et al., 2011). Furthermore, it could be proven that mice are more susceptible to tumorigenesis containing heterozygous beclin 1, a protein, which regulates macroautophagy. If it is overexpressed, tumor development is inhibited, on the other hand, cancer cells utilize autophagy for survival (Boutouja et al., 2017). Autophagy recycles ATP, which is needed by cancer cells in higher amounts, and it could be shown that the inhibition of autophagy genes by treatment with 3-methyladenosine or Atg7-knockdown activates apoptosis in different tumor cell lines, like prostate and colon cancer cells (Bhutia et al., 2013; Li et al., 2018). These results lead to two different strategies in cancer therapy. On the one hand, autophagy is induced, leading to enhanced tumor suppression; on the other hand, autophagy is inhibited and can induce apoptosis.

### HDAC Inhibitors Affecting Protein Quality Control Systems in Cancer Treatment

As mentioned in section “The Role of HDACs in Epigenetics and Cancer Cells,” HDACi cause hyperacetylation of histones which is one reason for the induction of apoptosis. As autophagy and apoptosis functionally counteract each other in tumor cells (see section “The Role of Protein Quality Control Systems in Cancer Cells”), one could assume that autophagy is inhibited during treatment with HDACi (Gump and Thorburn, 2011). However, it has been demonstrated that administration of HDACi can also induce autophagy, leading to the paradox situation that autophagy has beneficial effects in the treatment of cancer cells and even facilitates tumor suppression (Zhang et al., 2015). The effect of promoting cell survival or cell death is dependent on the cell type and genetic predisposition of the tumor, as well as the duration and dose of the HDACi. There are many examples described in the literature, one of them is the pan-HDACi panobinostat. On the one hand, panobinostat can inhibit autophagy by increasing the level of acetylation of autophagy-related gene products, for instance ATG7. The acetylation causes a repression of ATG7, which leads to a promotion of apoptosis and decreased autophagy in myeloid leukemia cells (Stankov et al., 2014). On the other hand, it has been reported that autophagy is induced in panobinostat-treated Eμ-myc lymphoma cells, the c-myc gene is here driven by the IgH enhancer. Thereby, an apoptotic protease activating factor 1 (Apaf-1) or caspase-9 deletion has been reported. This deletion causes an apoptosome inactivation and thus a suppression of apoptosis (Mrakovcic et al., 2017, 2018). Furthermore, it has been demonstrated that HDACs themselves can induce autophagy. For example, HDAC6 can induce autophagy as a result of an impaired UPS (Kaliszczak et al., 2018). It binds polyubiquitinated proteins and plays an essential role for the fusion of autophagosomes with lysosomes.

It seems to be a drawback that pan-HDACi not only inhibit histone deacetylation in the nucleus, but also a variety of proteins which can be found in virtually all cellular compartments. However, a greater understanding of the control and homeostasis mechanisms of HDACs is required to enable more effective application of HDACi for the treatment of specific tumor cell types.

## HDAC Inhibitors in Preclinical Studies

### HDAC Inhibitors Affecting Protein Quality Control Systems

As existing HDACi are mostly pan-HDACi, they do not entail satisfactory specificity. Accordingly, it is of great interest to develop new and more specific inhibitors (see also section “Novel Strategies of HDAC Inhibitors Affecting Protein Quality Control Pathways”). HDAC6 is a potential selective target due to its unique molecular structure with two catalytic domains and its localization in the cytoplasm (Li et al., 2018). Critical substrates with a role in PQC include p300 and HSP90 (Cosenza and Pozzi, 2018). Both are known to play a crucial role in tumorigenesis, showing the importance for the development of specific HDAC6 inhibitors. Tubastatin A is an example for an established HDAC6 inhibitor, often used in pre-clinical studies (Wang et al., 2016). However, a more specific HDAC6 inhibitor (marbostat-100) has been developed and was published in 2018 with a *K*_*i*_-value of 0.7 nM (Sellmer et al., 2018). In comparison, the value of the most common used selective HDAC6 inhibitor tubastatin A is 10-times higher. A preferred substrate of HDAC6 is α-tubulin; the reverse reaction is catalyzed by the α-tubulin acetyltransferase ATAT1. The deacetylated α-tubulin polymerizes with β-tubulin to form microtubules, components of the cytoskeleton, which play an important role in DNA segregation during mitosis. Inhibition of HDAC6 with marbostat-100 results in hyperacetylated α-tubulin. The specificity of this HDAC6 inhibitor was determined by comparing the enrichment of acetylated histone H3 in marbostat-100 treated cells with entinostat (MS 275) treated cells. It is an HDAC1 and HDAC3 specific inhibitor in phase II clinical studies. This enrichment of acetylated histone H3 could also be detected using the FDA-approved pan-HDACi panobinostat (LBH589) (Grünstein, 2018). It has been demonstrated in different human cell lines and in mice that marbostat-100 is considerably more specific than panobinostat, led more efficiently to hyperacetylation of α-tubulin and displayed only minor proteolytic effects on the target enzyme HDAC6 (Grünstein et al., 2019).

In analogy to marbostat-100, the application of the established HDAC6i tubastatin A led to hyperacetylation of α-tubulin. However, upon oxidative stress, tubastatin A activated the heat shock transcription factor 1 (HSF1) leading to the upregulation of the molecular chaperones HSP70 and HSP25 and increased cell survival (Leyk et al., 2017). Upon proteasomal stress, HDAC6 could initiate autophagy, as it is involved in the transport of ubiquitinated proteins along microtubules (Leyk et al., 2015). This influence of the PQC system has been observed in some preclinical studies of HDACi, which are described below in section “HDAC Inhibitors in Combination With Proteostatic Drugs.”

Two further examples for inhibitors that influence molecular chaperones as well as autophagy are trichostatin A and sodium butyrate. Both can affect the chromatin structure at the site where the gene for HSP70 is located (Chen et al., 2002). HSP70 is a molecular chaperone supporting the folding of many newly synthesized proteins and can recognize the KFERQ motif in proteins resulting in CMA.

In addition to its impact on molecular chaperones and consequently on CMA, another mechanism of action of trichostatin A is known. It has been shown that trichostatin A enhances the ubiquitination of the HAT p300, resulting in its proteasomal degradation. Since it is a co-activator of the expression of the NADPH oxidase 4 (Nox4) (Hakami et al., 2016), an important factor in angiogenesis, cancer cells suffer from oxygen and nutrient deficiency due to its reduced expression in trichostatin A treated cells. Thus, trichostatin A constitutes a paradigm in its ability to impact on molecular chaperones, the UPS and autophagy.

Another example for an inhibitor affecting the ubiquitin-proteasome system is MC1568 (Table 1). It is a class IIa selective HDACi that increases the specific sumoylation of HDAC4. Sumoylation is a PTM using “small ubiquitin-related modifier” (SUMO) to label the target protein and can direct it to different pathways. MC1568 induced HDAC4 down-regulation by increasing its specific sumoylation followed by activation of proteasomal degradation pathways. MC1568 alters not only the pattern of PTMs and activates the degradation of substrates *via* the UPS, but also changes epigenetic pathways that may be affected by HDAC4 (Scognamiglio et al., 2008).

**TABLE 1:** Preclinical studies of HDACi and compounds affecting PQC in combinatorial treatment.

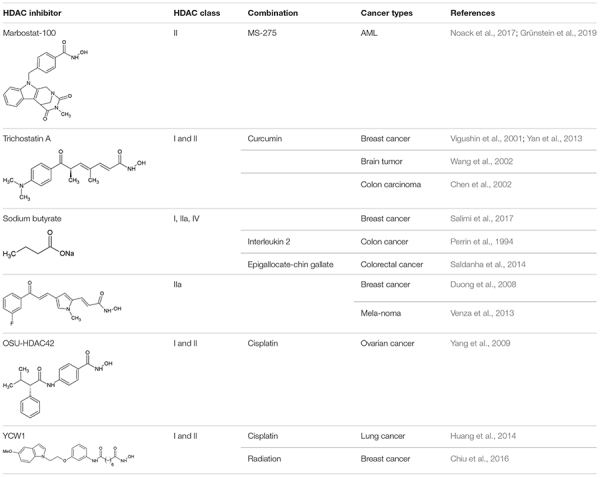

Besides trichostatin A, there are other HDACi known to induce autophagy. For instance, OSU-HDAC42 led to downregulation of Akt/mTOR signaling and the induction of the ER stress response to induce autophagy (Liu et al., 2010). On the contrary, triple negative breast cancer cells treated with the HDACi YCW1 showed a downregulation of BNIP3 (Bcl-2/adenovirus E1B 19 kDa protein interacting protein 3) resulting in autophagic cell death. Similarly, treatment of mice with YCW1 led to a decline in lung tumor growth (Huang et al., 2014).

### HDAC Inhibitors in Combination With Proteostatic Drugs

The fact that HDACi can influence PQC systems led to a novel strategy where HDACi are used in combination with drugs modulating PQC systems, i.e., PI or modulators of autophagy.

In a study conducted in 2017, trichostatin A was tested in combination with the PI bortezomib for the treatment of ovarian cancer cells and displayed an inhibition of the proliferation of A2780 cells inducing apoptosis. Furthermore, similar results were shown in A2780T cells that are resistant to cytostatic taxanes (Jin et al., 2017). In another attempt, a combination of sodium butyrate was tested with the PIs MG115, MG132, PSI-1, PSI-2, or epoxomicin in human CRC cells (SW48, SW1116, and SW837). In these studies, additive and synergistic anticancer effects, namely growth inhibition and apoptosis, were observed in combination with all tested PIs (Abaza, 2010).

Cell death induced by accelerated autophagy in cancer cells has been shown to be effective in a combined treatment with HDACi and an inducer of autophagy. In Burkitt lymphoma and lymphocyte cell lines, VPA induced autophagosome formation and increased autophagy led to an autophagy-mediated cell death in combination with an mTOR inhibitor (Dong et al., 2013). This experiment has been carried out with the mTOR-specific inhibitor temsirolismus, but as OSU-HDAC42 is known to act as an mTOR inhibitor, it is suspected that OSU-HDAC42 would show similar results (Liu et al., 2010). Furthermore, it has been demonstrated that the protein levels of HDACs, especially HDAC6, are reduced by autophagy after treatment with the HDACi AR42 (OSU-HDAC42). The combination of this HDACi with the kinase inhibitor pazopanib in melanoma cells demonstrated an inhibition of the ATPase activity of the molecular chaperones HSP90 and HSP70. In this setting, HDAC6 could activate HSP90 by deacetylation and the inhibition was enhanced by combined treatment (Booth et al., 2017). Using YCW1 (Table 1) in combination with radiation also demonstrated increased cell death in cancer cells due to ER stress and the induction of autophagy (Chiu et al., 2016). In addition, preclinical studies revealed an enhanced cisplatin effect against YCW1-treated non-small cell lung cancer (NSCLC), whereas cisplatin causes mitochondria-mediated apoptosis (Huang et al., 2014). This highlights again the discrepancy that cancer cells sometimes utilize autophagy to their advantage and sometimes inhibit the autophagic pathway.

Protein ubiquitination can be also mediated by cullin-ring E3 ligases (CRLs), which have to be activated by neddylation with NEDD8 (neural-precursor-cell-expressed developmentally down-regulated 8), another protein similar to ubiquitin acting as PTM (Enchev et al., 2015). Neddylation with NEDD8 is achieved by NEDD8-activating enzymes (NAEs), which are druggable enzymes. It has been shown in several studies that NAE-inhibitors, for instance pevonedistat (MLN4924), can act with other anti-cancer agents including bortezomib in a synergistic manner in MM (Gu et al., 2014). In a different pre-clinical study, it has been demonstrated that the NAE inhibitor pevonedistat acts synergistically with the HDACi belinostat in various AML cell types, especially those with reciprocal effects on homologous recombination (HR) and non-homologous end-joining (NHEJ) mechanisms (Zhou et al., 2016).

### HDAC Inhibitors and Bromodomain Inhibitors

There are 61 bromodomains known in the human proteome, integrated in 46 proteins. All of them have a conserved left-handed bundle of four α-helices linked by flexible and variable loops. The best-known proteins containing a bromodomain are part of the bromodomain and extra terminal family (BET). This BRD family is characterized by the presence of two tandem bromodomains (BD1 and BD2) at the *N*-terminus, an extra terminal domain (ET), and a C-terminal domain (CTD). They play a crucial role in cancer cells, especially in cell proliferation by regulating the expression of oncogenes, for instance c-MYC or nuclear factor κ light chain enhancer of activated B cells (NF-κβ)-dependent genes (Pérez-Salvia and Esteller, 2017).

It has been demonstrated that hyperacetylation of proteins induced by HDACi can result in amyloid-like protein aggregation. This can lead to a reduction of the proteolytic capacity of the UPS, increased autophagy and downregulated translation, summarized as proteostatic failure (Olzscha et al., 2017). Similarly, it has been observed that trichostatin A induced a dramatic increase of the acetylation of tau proteins, which aggregation can be seen under pathological conditions as one of the underlying causes for Alzheimer’s disease (AD) (Cohen et al., 2011). The increase of acetylated tau levels and resulting aggregation is also shown in tubastatin A treated oligodendrocytes, and an alteration of the cell morphology was observed containing a reduced microtubule binding activity of tau (Noack et al., 2014). As described above, tubastatin A is an HDAC6-specific inhibitor; consequently, HDAC6 plays an important role in the cytotoxic accumulation of protein aggregates which may explain the higher level of aggregated tau proteins (Boyault et al., 2007b). Marbostat-100 also inhibits HDAC6, the treatment may have similar effects on the acetylation of the tau protein; however, it remains unclear whether it has an effect on aggregation.

The potential of HDACi-induced aggregation raises the question, how this knowledge can be utilized for benefits in cancer therapy, at the same time preventing adverse side effects and suppressing aggregation. It has been demonstrated that the bromodomain-containing proteins CBP and p300 are involved in the formation of protein aggregates after treatment with HDACi and their depletion results in a reduction of aggregation. This opens a new strategy for cancer treatment without the formation of aggregates. Bromodomain inhibitors are small proteins which can block the binding of bromodomain-containing proteins to acetylated residues and therefore have the potential to reverse the aggregation-induced cytotoxicity and restore proteostasis (Olzscha et al., 2017).

The first published BET inhibitor was (+)-JQ1 tested in NUT (nuclear protein in testis) midline carcinoma cells. NUT can fuse with the bromodomain BRD4 forming the oncoprotein BRD4-NUT, which plays an important role in the differentiation and proliferation of cancer cells. (+)-JQ1 acts as a competitor binding at the acetyl-lysine binding motif and prevents the formation of the fusion protein and the resulting proliferation (Filippakopoulos et al., 2010). It was then tested in many other cancer types, for instance glioblastoma (Cheng et al., 2013), colon cancer (McCleland et al., 2016), lung cancer (Lockwood et al., 2012), Burkitt’s lymphoma (Mertz et al., 2011), and MM (Soodgupta et al., 2015). It led to downregulation of c-MYC, an oncogene responsible for altered transcription and proliferation and showed synergistic effects with the HDACi mocetinostat (Borbely et al., 2015). However, it has never reached a clinical trial, due to its short half-life; therefore, analogs of (+)-JQ1 with a longer half-life were synthesized, one of them is called CPI203 (Alqahtani et al., 2019). It has shown some success in bortezomib-resistant mantle cell lymphoma (Moros et al., 2014) and MM cells (Díaz et al., 2017) in combination with the immunomodulator lenalidomide, whereby reduced c-MYC-levels leading to a downregulation of IRF4 (interferon regulatory factor 4). IRF4 is a transcription factor, which is necessary for the survival of lymphoma and myeloma cells, leading to an induction of apoptosis in these cells. I-BET151 is another BET-inhibitor, which also represses c-MYC in myeloma cells (Chaidos et al., 2014). As a pan-BET inhibitor, it also displayed anti-cancer effects in other types of cancers, for example in medulloblastoma cells by suppressing the Hedgehog-activity or in NUT midline carcinoma (Long et al., 2014).

Other examples where bromodomain inhibitors can influence proteostasis in combination with HDACi are CBP and p300. These transcription modulators are not only bromodomain-containing proteins, they also act as histone acetyltransferases. Thus, they recognize lysine acetylation and may cause further acetylation in histones leading to a relaxation of DNA and an activation of transcription. Two p300/CBP-specific bromodomain inhibitors, I-CBP112 and SGC-CBP30, were investigated in preclinical studies, I-CBP112 for leukemia and prostate cancer (Picaud et al., 2015) and SGC-CBP30 in MM (Hay et al., 2014). I-CBP112 activates the HATs CBP and p300 resulting in a repression of the proliferation in cancer cells (Zucconi et al., 2016). SGC-CBP30 can suppress IRF4 in myeloma cells (Conery et al., 2016). However, currently it is not brought into clinical trial, as it displays a short half-life. Another inhibitor which targets non-BET bromodomains as well as BET-bromodomains is bromosporine (Theodoulou et al., 2016). This pan-BDi reduced the formation of protein aggregates only slightly after HDACi treatment (Olzscha et al., 2017).

There is still an interest in developing more specific and more efficient HDACi for cancer treatment. Most of the current HDACi influence several pathways in the cells, including the PQC system. This can be used by combining HDACi with other cancer treatments, like radiation, PI, bromodomain inhibitors, autophagy- and chaperone-modulating agents. On the other hand, there are also new HDACi broadening the spectrum of molecular actions and therefore their monotherapeutic use has to be reconsidered as a treatment option.

### Novel Strategies of HDAC Inhibitors Affecting Protein Quality Control Pathways

Various novel HDACi have undergone pre-clinical and clinical studies over the past 5 years, both HDACi which target specific HDAC classes and HDACs which can be considered as pan-HDACi. CXD101 is a novel class 1-selective HDACi and has shown effects in some hematological malignancies (Eyre et al., 2019). The observed high levels of the proteasomal shuttling factor HR23B indicate a positive outcome, resembling the results of pan-HDACi (Khan et al., 2010; New et al., 2013). In fact, it has been demonstrated that also class I HDACi are able to induce protein aggregation in human cells (Olzscha et al., 2017). As described above, the induced protein aggregation may contribute to the overall cytotoxicity exerted by HDACi and their success in hematological malignancies. Since CBP/p300-specific bromodomain inhibitors are able to partially abrogate this effect (Olzscha et al., 2017), it is likely that the aggregation indirectly affects nuclear proteins and therefore modulates epigenetic regulation in cells. Interestingly, a clinical trial of CXD101 in combination with the tissue-agnostic drug pembrolizumab for relapsed or refractory diffuse large B-cell lymphoma (PLACARD, NCT03873025) is underway, taking the levels of PD-L1 (programmed death-ligand 1) in PD-1 positive cells into account (see also section “Evaluating Alterations of PQC Systems: Precision Medicine Upon HDAC Inhibitor Treatment”).

As mentioned above (section “HDAC Inhibitors in Combination With Proteostatic Drugs”), a common strategy is to apply HDACi in combination therapy with other anti-cancer drugs. However, this strategy faces several problems, including incompatibilities, pharmacokinetic problems when reaching different compartments and unexpected interactions, which may alleviate the activities, but also lead to an increased possibility to generate undesired cytotoxic effects (de Lera and Ganesan, 2016). To overcome some of the problems, several chimeric HDACi have been developed. They consist of hybridized functional groups of an HDACi structure and the respective different group to inhibit or bind to a second target (Nepali et al., 2014). For instance, the inhibitor fimepinostat (CUDC-907), which is a dual HDACi and phosphatidylinositol-3-kinases (PI3K) (Gunst et al., 2019) was tested in a trial in patients with lymphoma (NCT01742988). A striking example of this strategy, which affects epigenetic outcome and PQC pathways, is the generation of chimeras between HDACi and bromodomain inhibitors (BDi). Bromodomains cannot only “read” acetylation marks on proteins, they can also act synergistically with HDACs to guide them to the respective protein and remove the acetylation mark (Olzscha et al., 2015). Many promising examples of BDi have been generated, targeting several bromodomain-containing proteins, including CBP/p300, affecting both chromatin and interacting proteins, including p53 (Picaud et al., 2015). Several chimeric compounds consist of the functional groups of pan-HDACi such as SAHA and different BDi including the BET inhibitors JQ1 and I-BET295 (Atkinson et al., 2014), as well as BRD-4 specific inhibitors (Amemiya et al., 2017). Besides the established functions as epigenetic modulators, some HDACs and bromodomain-containing proteins (BRDs) exert their activities also in the cytoplasm, affecting crucial PQC pathways. Effects of HDACs and BRDs on the protein degradation machinery demonstrate that these mechanisms contribute to the overall cytotoxicity of the single substances or their chimeras (New et al., 2013; Olzscha et al., 2017).

A novel HDACi which affects proteostasis is MPT0G413, a selective HDAC6 inhibitor. This inhibitor did not only inhibit the growth in MM cells, the combination of MPT0G413 and bortezomib enhanced also polyubiquitinated protein accumulation and synergistically reduced MM viability, showing increased caspase-3, caspase-8, and caspase-9 levels (Huang et al., 2019). Since it is an HDAC6 inhibitor, the effects are likely to reflect disturbances in PQC pathways and therefore only indirectly affect epigenetic features in the nucleus.

## Clinical Studies: HDAC Inhibitors in Combination With Modifiers of Protein Quality Control

The knowledge about epigenetics has exploded over the past few years, highlighting its importance in crucial functions in the cell such as gene silencing, DNA methylation and histone modification. The observation of aberrant hypermethylation on CpG-rich promoter regions, histone modification, non-coding RNA modification and other epigenetic changes in cancer cells established the research field of the “cancer epigenome” and spurred efforts to investigate appropriate therapies in this newly defined field. During the past few years it has become increasingly apparent that neoplastic cells have a selective advantage not only due to mutations, but also provided by epigenetic changes (Arrowsmith et al., 2012). Histone modification such as HDAC overexpression or altered acetylation levels have been found in prostate, gastric, colorectal, cervical and endometrial cancer (Glozak and Seto, 2007), see also section “HDACS in Epigenetics and Protein Quality Control Systems.” In addition, a negative correlation between HDAC overexpression and overall survival has been described in pancreatic, breast, colorectal, gastric, lung, liver cancer and melanoma (Weichert et al., 2008a; West and Johnstone, 2014; Mottamal et al., 2015; Sarkar et al., 2015). In particular, broad ranges of hematological malignancies appear to be influenced by HDAC alterations. For instance, high expression of HDAC1, 2 and 6 are persistent in patients suffering from CTCL (Marquard et al., 2008). As hematological malignancies were described to be especially sensitive to HDACi therapy, a wide set of clinical trials exploited the research field of monotherapy of HDACi in hematological malignancies with partly successful and promising results (Cashen et al., 2012; Fukutomi et al., 2012; Kirschbaum et al., 2012, 2014; Younes et al., 2012; Platzbecker et al., 2014). Until now, four HDACi are approved by the FDA, as described in the introduction. Although HDACi monotherapy has been described to show promising effects on these types of tumors, further investigations in solid tumors were disappointing, as a large-scale use of HDACi is hampered due to a lack of detailed understanding in molecular mechanisms of HDACi. Several clinical trials revealed that monotherapy with HDACi only showed limited success in solid tumors (Qiu et al., 2013). In addition, some clinical studies had reportedly severe side effects using HDACi in monotherapy, another reason why they were discontinued. Hence, toxicity profiles need to be considered in future clinical trials, especially when combining HDACi with other agents (Subramanian et al., 2010). However, HDACi revealed to function synergistically with a range of other anticancer agents such as immune checkpoint inhibitors, platinum-based chemotherapeutics or tyrosine kinase pathway inhibitors in pre-clinical and clinical studies. Therefore, combination of HDACi with other cancer therapeutics may represent an important direction to enhance their anticancer efficacy and show their full therapeutic potential (Singh et al., 2018; Suraweera et al., 2018).

At present, various clinical trials are in progress, testing different HDACi for both hematological and solid tumors in either mono- or combined therapy regimens (Table 2).

**TABLE 2:** HDACi in combination with inhibitors of protein quality control systems in clinical trials.

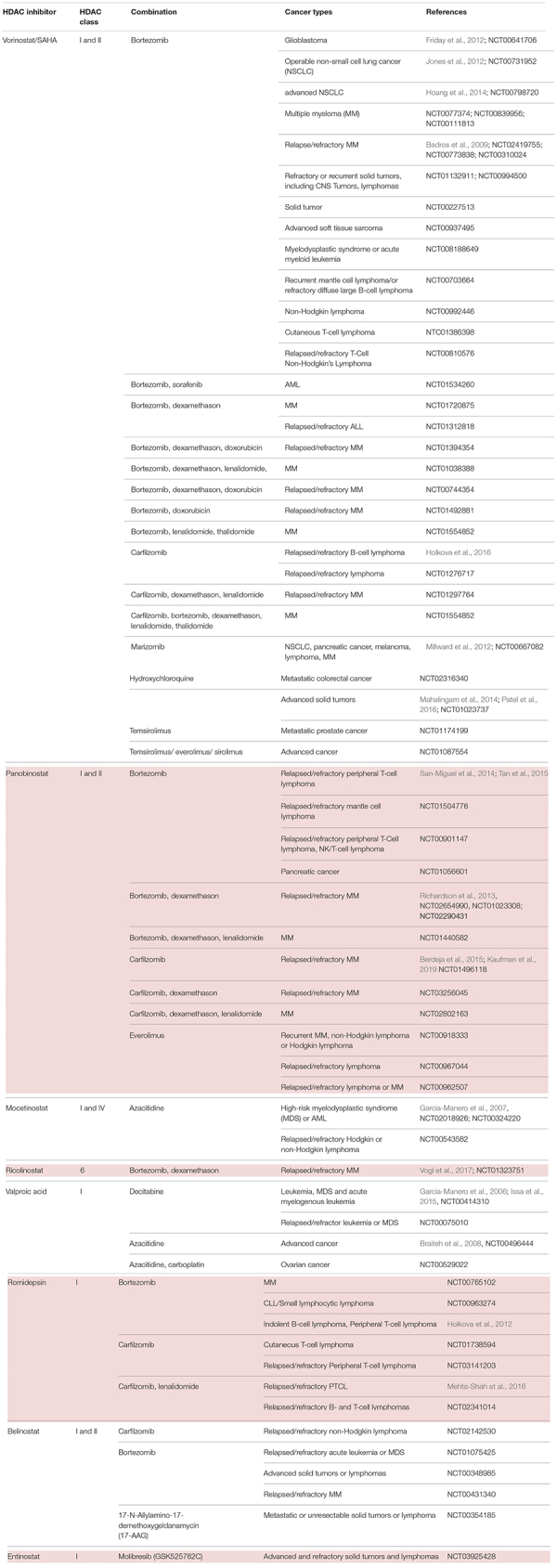

### HDAC Inhibitors and Proteasome Inhibitors

Cancer cells are highly proliferative and show an extensive protein turnover and thus rely heavily on proteasomal degradation of abnormal or mutant proteins (Adams, 2004). Therefore, it can be argued that cancer cells are more dependent on functioning PQC systems such as the UPS, and autophagy than non-transformed cells (Goldberg, 2007). Indeed, several preclinical studies established that proteasome inhibition has a more severe effect on malignant cells than on normal cells (An et al., 1998; Masdehors et al., 2000; Hideshima et al., 2001; LeBlanc et al., 2002). Thus, proteasome inhibition would overload the cancer cell with protein material and accumulation of ubiquitinated proteins, finally causing cell death (Adams, 2004).

First attempts on using bortezomib, as a single agent PI, showed success in the treatment of relapsed and refractory MM. Various preclinical and clinical trials provided data on significant benefit in patients’ respond and outcome (Orlowski et al., 1998; Hideshima et al., 2001; LeBlanc et al., 2002; Richardson et al., 2005a). The Assessment of Proteasome inhibition for EXtending remissions (APEX) trial confirmed significant benefit in the bortezomib group over the patients treated with dexamethasone in patients with relapsed MM (Richardson et al., 2005b). These findings laid the foundation for the approval of bortezomib by the FDA in 2003. Although thrombocytopenia and peripheral neuropathy were the most frequently associated dose limiting toxicities, the FDA authorized bortezomib for the use in relapsed and refractory myeloma patients who showed no response to two or more prior therapies (Field-Smith et al., 2006). Accordingly, two other substantial drug discoveries were found to have a beneficial effect on MM patients: the immunomodulatory drugs thalidomide and lenalidomide (Dimopoulos et al., 2007; Rajkumar et al., 2008). However, despite all the promising new drug developments and outcomes, a number of patients refractory to prior use of bortezomib, thalidomide, or lenalidomide still only showed poor responses (Kumar et al., 2017). As MM cells have been described to possess an abnormal acetylome, another approach to this group of patients was the implementation of HDACi (Mithraprabhu et al., 2014). In fact, data of preclinical studies demonstrated an anti-proliferative effect of vorinostat, romidepsin, dacinostat and panobinostat resulting in apoptosis of MM cells (Catley et al., 2003; Khan et al., 2004; Campbell et al., 2010; Ocio et al., 2010; Sanchez et al., 2011; Holkova et al., 2012). However, when transferring single agent use of HDACi into clinical trials, only limited effect on MM cells was noted (Richardson et al., 2008; Niesvizky et al., 2011).

Eventually, several preclinical studies postulated synergistic effects of HDACi and proteasomal inhibition, paving the way of combinational therapy of these two agents (Pei et al., 2004; Campbell et al., 2010). The best characterized and coherent explanation of the synergy between PI and HDACi is the dual inhibition of the proteasome and aggresome pathway (Hideshima et al., 2005; Catley et al., 2006). Targeting both of the degradation pathways with bortezomib and HDACi in tumor cells would exponentiate their effect and result in greater accumulation of polyubiquitinated proteins, increased cellular stress and apoptosis. More specifically, despite the fact that proteasome inhibition results in accumulation of ubiquitinated proteins and cell death, malignant cells have shown to evade this life-threatening end by an alternative pathway. Here, malignant cells form aggresomes, engulfing the polyubiquitinated proteins to transport them with the help of HDAC6 *via* microtubules (Ouyang et al., 2012). Disruption of this alternative pathway was reported using both nonselective and selective HDACi through HDAC6 inhibition thus synergizing with bortezomib and inducing cells to undergo apoptosis in multiple hematologic and epithelial malignancies (Catley et al., 2006; Nawrocki et al., 2006; Heider et al., 2008). In the same vein, the beforementioned observation that pan-HDACi treatment in clinical concentration of human cells led to the formation of amyloid fibrils gave a further proof that HDACi may act synergistically on PQC pathways (Olzscha et al., 2017). On account of the described synergy, dual inhibition could exploit full therapeutic potential of both proteasome and HDAC inhibition.

A hallmark of MM cells is the production of abundant amounts of immunoglobulin which must either be properly folded or degraded. Accordingly, dual disruption in protein degradation seemed to be especially effective in these types of cancer cells (Lee et al., 2003; Obeng et al., 2006). This assumption was substantiated by preclinical data, indicating a combination of PI and HDACi to be an attractive and novel strategy for the treatment of MM (Table 2).

Preliminary data from phase I, II and III studies evaluated success in the treatment regime of panobinostat or vorinostat as HDACi plus bortezomib in patients with relapsed or refractory MM (Badros et al., 2009; San-Miguel et al., 2013). Subsequently, combinational therapy was implemented into clinical settings. Patients with relapsed or refractory MM were examined on the effect of HDACi combined with proteasome inhibition. On the one hand, the phase II VANTAGE trial analyzed vorinostat plus bortezomib (Dimopoulos et al., 2013), whereas on the other hand, the phase II PANORAMA 1 trial tested the combination of panobinostat plus bortezomib and dexamethasone in patients with relapsed and refractory MM (San-Miguel et al., 2011). Despite the fact that VANTAGE displayed prolonged progression-free survival (median PFS of 7.6 vs. 6.8 month) when combining vorinostat and bortezomib, clinical relevance needed to be further examined. The PANORAMA 1 trial was able to show modest overall survival benefit when combinational therapy of panobinostat, bortezomib and dexamethasone was applied (median PFS of 12 vs. 8 month). This led to the approval of panobinostat in 2015 by the FDA. The results of this therapeutic approach were evaluated on bortezomib-refractory patients in the PANORAMA 2 trial (Richardson et al., 2013). According to this trial, it has been proposed that combinational treatment of panobinostat, bortezomib and dexamethasone recaptures response in 34.5% of pre-treated, bortezomib-refractory MM patients. In summary, both results of PANORAMA 1 and 2 are partly coherent with preclinical studies and elucidate considerably the role of panobinostat in combination with bortezomib and dexamethasone, especially in patients with relapsed or bortezomib refractory MM. Furthermore, the results hypothesize HDACi to sensitize patients with bortezomib-resistant MM. Despite the clinical benefit of this combined agent regime, it harbors the danger of poor side effects. Especially the overlapping toxicity profiles make this regime rather toxic. A grade 3 – 4 thrombocytopenia (67%) and gastrointestinal toxicity (diarrhea 25%) indicate a poor safety profile. In order to optimize the safety profile, combinations of panobinostat with second-generation PI were tested at different doses and schedules. Carfilzomib is one example of a second-generation PI and obtained approval by the FDA for the treatment of relapsed and refractory MM, in patients who were given at least two prior therapies (Groen et al., 2019). Combination of panobinostat and carfilzomib was tested in a phase I/II clinical trial in patients with relapsed or refractory MM with promising response rates and an acceptable safety profile (ClinicalTrials.gov identifier: NCT01496118) (Berdeja et al., 2015).

Another idea to address the rather toxic combination of pan-HDACi with PI was to substitute pan-HDACi with class-selective HDACi. In contrast to pan-HDACi, selective class I HDACi rarely induce thrombocytopenia, thus seeming to be more suitable agents. As the key mechanism underlying the synergistic effect of HDACi and PI was mainly explained by HDAC6-dependent aggresome function, it can be argued that class-specific HDACi may still synergize with PI without having a poor safety profile. Therefore, an isoform selective HDAC6 inhibitor, ricolinostat, was introduced in the combinational treatment regime with bortezomib and dexamethasone in relapsed or refractory MM patients (Vogl et al., 2017). This phase I/II study demonstrated that combinational therapy with an isoform selective HDACi shows less severe gastrointestinal, hematologic, and constitutional toxicities in comparison to non-selective HDACi. This raised the idea to test the novel combinational regime in different malignancies. It has been demonstrated that class I HDACi mocetinostat/MGCD0103 has a potent antiproliferative activity in Hodgkin lymphoma (HL) cell lines in an HDAC6-independent manner (Buglio et al., 2010). Regarding these results, mocetinostat was especially interesting to investigate further, as it is a class I HDACi with no effects on HDAC6 (Fournel et al., 2008). The generated data demonstrated that inhibition of class I HDAC by mocetinostat results in an adequate induction of cell death in HL cell lines. On account to that, a broader inhibition of HDACs, including HDAC6, is not needed for a sufficient antiproliferative effect *in vitro*. Furthermore, they were able to show a synergistic effect of mocetinostat with PI. Mocetinostat induced the expression of various inflammatory cytokines resulting in the activation of NF-κB, which in turn mitigated the killing effect of mocetinostat on tumor cells. As PIs inhibit NF-κB activation, this novel combination would explain how PIs enhance mocetinostat activity, independent of HDAC6. A following phase II trial tested mocetinostat, a class I/IV HDACi for relapsed HL, whereby 85 mg were administered three times per week. 14 of 51 patients (27%) treated with mocetinostat had a complete or partial response whereas only one patient out of 25 (4%) had a partial response on pan-HDACi vorinostat. Single agent use of mocetinostat also induced a reduction in tumor size in more than 4/5 of patients (Younes et al., 2011). Collectively, these data suggest the potential and clinical value of class-specific HDACi in patients with HL. Combination of panobinostat and bortezomib in patients with relapsed or refractory PTCL shows encouraging activity, however displaying a relatively high number of adverse events with 10 out of 25 patients (40%) (Tan et al., 2015).

Noticeably, HDACi and PI have been tested and analyzed in a variety of hematological malignancies. However, their effects are not well investigated in solid tumor malignancies. First attempts in investigating the safety and efficacy of vorinostat and bortezomib were tested 2012 in NSCLC. Here, they examined the two-agent use as induction therapy with an adjacent surgery in patients with NSCLC (Jones et al., 2012). The obtained results showed a decrease in metabolic activity in the tumors. However, due to the short duration of induction treatment, no significant change in tumor size has been observed. A following phase II study testing vorinostat and bortezomib as third-line therapy in patients with advanced NSCLC was terminated at its first temporary analyses due to a lack of anti-tumor activity (Hoang et al., 2014). Nonetheless, they highlighted the relevance of potential biomarkers predicting drug activity and thus driving clinical development.

In another setting, HDACi and PI have been tested in glioblastoma multiforme (GBM) cells, as promising preclinical studies proposed activity against GBM cell lines and glioma models (Eyüpoglu et al., 2005; Ugur et al., 2007; Yin et al., 2007). However, this result was not confirmed in clinical trials. A phase II trial of bortezomib in combination with vorinostat in recurrent glioblastoma had disappointing results and was clinically ineffective (Friday et al., 2012). It should be considered that unlike vorinostat, bortezomib cannot pass an intact blood-brain barrier (BBB) and thus may be the reason for an unsatisfactory result. Since other PIs such as marizomib can cross the BBB, it would eventually be a more beneficial combination with an HDACi in treating tumors beyond the BBB (Gozzetti and Cerase, 2014).

Overall, HDACi in combination with PI showed synergistic effects, which could be validated in several phase I trials in different tumor entities. However, there is still an unmet need of further investigation on molecular mechanisms underlying the combinational treatment regime and especially of the development of predictive biomarkers. These biomarkers (see also section “Evaluating Alterations of PQC Systems: Precision Medicine Upon HDAC Inhibitor Treatment”) would allow clinicians to stratify patients who would benefit from the treatments (Table 2).

### HDAC Inhibitors and Modulators of Autophagy

Another approach, going for the same train of thought as seen in the combinational regime of HDACi and PI is the substitution of PI with autophagy inhibition (Table 2). As outlined in section “The Role of Protein Quality Control Systems in Cancer Cells,” autophagy represents a hallmark of the PQC as the proteasome does. Preclinical studies showed a context-dependent effect of autophagy in different states of malignant pathogenesis. As outlined in the section about pre-clinical studies, autophagy has a protective function in premalignant cells. While preventing defective cells from proliferating, it hampers the acquisition of additional mutations that would even promote tumor development. However, looking at advanced cancer cells, autophagy can promote mechanism for oncogenesis. Here, it enhances cell survival under stressful conditions in the tumor microenvironment, such as hypoxia and nutrient deprivation (White, 2012). Besides the already existing endogenous stress, autophagy is even further promoted by anti-cancer treatment, leading to additional protection of tumor development (Janku et al., 2011). This could give a rationale for the poor therapeutic efficacy, as the survival of malignant cells is maintained through autophagy (Carew et al., 2007a, 2012; Amaravadi et al., 2011). Recent investigations confirm the diminished effect of therapies due to autophagy, promoting cancer cell survival (Strait et al., 2002; Duan et al., 2005). Thus, inhibition of autophagy represents a novel strategy to augment cancer treatment efficacy.

HDACi have been described to induce autophagy in many clinical trials, but its full therapeutic potential is hampered due to the protective action of autophagy. Subsequently, disruption of autophagy would boost the pro-apoptotic and cytostatic effects of HDACi. In fact, this mode of action was confirmed by preclinical studies in models of imatinib-resistant chronic myeloid leukemia and colon cancer (Carew et al., 2007b, 2010). In 2014, Mahalingam et al. reported about a phase I clinical trial of the autophagy inhibitor hydroxychloroquine (HCQ) in combination with vorinostat in adult patients with advanced refractory solid malignancies (Mahalingam et al., 2014). In the majority of patients, no significant benefit was observed; only renal cell carcinoma patients had a dramatic and durable response to the novel combination of vorinostat plus hydroxychloroquine (HCQ). Besides the deficient results, the authors accentuated the need of predictive biomarker for assessing clinical sensitivity to autophagy inhibitors in order to optimize drug development. In 2016, Patel et al. revived the scheme and performed a single-arm expansion cohort to assess the efficacy, safety and effects on immunity of vorinostat and HCQ in patients with refractory metastatic CRC (Patel et al., 2016). Results implied no substantial benefit over other oral drug as survival showed to be comparable to other oral drugs for refractory CRC including regorafenib (Grothey et al., 2013). Despite this outcome, vorinostat plus HCQ had a favorable toxicity profile and can be discussed as an alternative treatment for refractory CRC. Subsequently, a randomized phase II trial of vorinostat and HCQ versus regorafenib (a tyrosinkinase inhibitor) is now open to enrollment. Alongside, another phase II trial exhibits the therapeutic benefit on vorinostat plus HCQ over regorafenib in chemo-refractory metastatic colorectal cancer. Results implied that survival of the two treatment regimens showed comparable survival (Arora et al., 2019).

### HDAC Inhibitors and HSP90 Inhibitors

**FIGURE 3:**
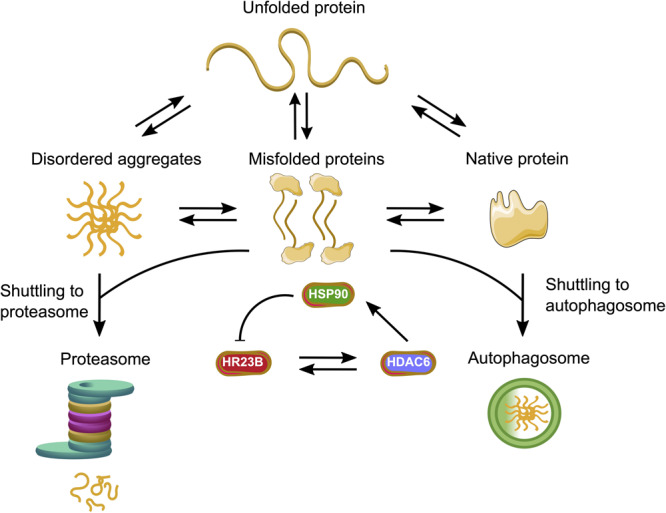
HDACs facilitate the interplay between the major protein quality control systems. HR23B as a proteasome shuttling factor can enhance the proteolysis of ubiquitinated proteins, whereas HDAC6 enables the autophagy process. HDAC6 can either bind HR23B with its BUZ domain or acetylate the molecular chaperone HSP90. The acetylated HSP90 can interact with HR23B and initiate a feedback loop.

Another interesting new idea was to combine HDACi with modulators of molecular chaperones. As outlined in section “The Role of Protein Quality Control Systems in Cancer Cells,” molecular chaperones and particularly HSP90 and HSP70 play not only a role in protein folding, but also in signal transduction and interact in several pathways to maintain cellular protein homeostasis and cell survival (Wiech et al., 1992). HSP90 has been described as a key player to stabilize proteins that are particularly important in cancer cells including BCR-ABL (BCR, breakpoint cluster region; ABL, Abelson), ERB-B2 (erythroblastic oncogene B), proto-oncogene B-Raf (BRAF), AKT Serine/Threonine Kinase (AKT), vascular endothelial growth factor receptor (VEGFR), FMS like tyrosine kinase 3 (FLT3), androgen and estrogen receptors, hypoxia-inducible factor (HIF-1α) and a constantly growing list, affecting a variety of cancer-related functions (Welch and Feramisco, 1982; Powers and Workman, 2006). In some cancerous cell lines, HSP90 has been found in much higher levels compared to normal cells (Kamal et al., 2003) and shows beneficial effects on many oncoproteins (da Rocha Dias et al., 2005; Shimamura et al., 2005). Preclinical studies demonstrated the anti-cancer effect of HSP90 inhibition and suggested its ability could affect several oncogenic signaling pathways simultaneously. Thus, it reduces the likelihood of the tumor acquiring resistance to any single therapeutic pathway and is a major advantage upon other agents (Banerji, 2009). However, HSP90 inhibitors such as 17-AAG have not reached clinical trials beyond phase III, due to minimal effects and toxicity, especially liver toxicity (Hyun et al., 2018). Therefore, the combination with other agents, for instance HDACi, could be a promising alternative, in order to reduce the effective concentration of HSP90 inhibitors. Following this hypothesis, HDAC6 and HSP90 are interactors as HDAC6 can deacetylate HSP90 (Boyault et al., 2007a; Figure 3). Adversely, when inhibiting HDAC6, HSP90 is present in a hyper-acetylated state, losing the bond with the co-chaperone p23 and finally its overall chaperone activity (Kovacs et al., 2005; Aldana-Masangkay and Sakamoto, 2011). This makes the synergistic use a promising anti-cancer treatment strategy (Table 2). Lung cancer has been described to be particularly susceptible to HSP90 inhibition. Overexpressed or mutant ERB-B2 and BRAF are often the driving force in lung cancer development. Interestingly, all of them are degraded with the assistance of HSP90, giving a rationale for the beneficial use of HSP90 inhibitors in lung cancer (Shimamura and Shapiro, 2008; Zismanov et al., 2014). This is now implemented into an ongoing clinical trial with 20 participants aiming to investigate the combined effect of HSP90 inhibitor and PI or HDACi on lung cancer cell fate and ER/Golgi homeostasis (NCT01270399).

### HDAC Inhibitors and Bromodomain Inhibitors

This novel combination treatment has not been well investigated in a clinical set-up to this point. Preclinical studies proposed synergistic effects in breast cancer (Rahmani et al., 2003). One phase I trial tested GSK525762C (molibresib besylate) and entinostat in patients with advanced or refractory solid tumors or lymphomas. Results are yet to come.

Overall, epigenetics has become an inevitable part of cancer research. Until now, HDACi have been shown and tested to synergize with a wide range of very different agents. Of particular interest is the synergistic use of HDACi with the inhibition of PQC systems. Herein, progress has been made, implicating that HDACi exhibit their anticancer activity through a multitude of pathways. However, there is still an unmet need for further investigations on detailed mechanistic action of HDACi. The therapeutic effect of HDACi not only depends on the cancer type, but on the stage of cancer, treatment dosage, the individual patient’s biological signature, and other factors. In order to boost the development of HDACi and PQC modulating agents these factors need to be considered. To achieve significant improvement in HDACi therapeutic outcomes, better patient selection and monitoring of biomarkers are strongly required.

## Evaluating Alterations of PQC Systems: Precision Medicine Upon HDAC Inhibitor Treatment

**FIGURE 4:**
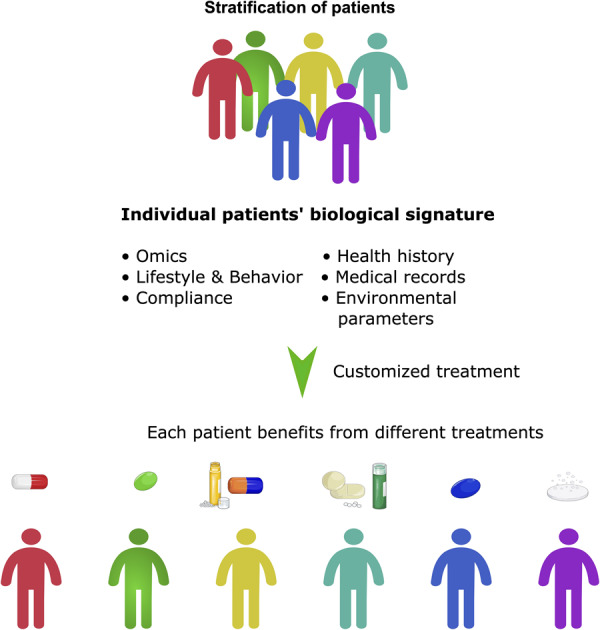
Precision medicine beyond stratification of patients. Precision medicine can be seen as a more holistic approach than personalized medicine. It takes many factors into account, not only the stratification of patients, but also their molecular signature, social and environmental factors and lifestyle. It often involves the application of pan-omic analyses and systems biology to determine the cause of an individual patient’s disease at the molecular level and then to utilize different targeted treatments.

The ability to cost-effectively sequence the human genome and epigenome to apply genetics in drug treatment has changed the approach of cancer treatment in a fundamental way and led to a revolution from “one-size-fits-all” therapy to a more precise therapy approach. It enabled to look at a patient as an individual comprising of a unique set of genes, proteins and environment and mainly formed the term of precision medicine (Figure 4). Here “the specific targeting of molecular abnormalities and the stratification of patients who respond to specific drugs” is in focus (Coyle et al., 2017). This personalized approach to stratify a patient group has attracted attention especially in cancer, where specific information about a patient’s tumor helps diagnose, treatment planning and making of prognoses. Thus, it seemed to be the next logical step in advanced cancer treatment. Alongside with our evolving knowledge about oncogenesis, cancer therapy must also be accompanied by a molecular understanding of both, genetic and epigenetic factors in cancer patients (Hanahan and Weinberg, 2000). Therefore, it is not only important to screen a set of patients’ genes, but it is equally important to predict whether this unique tumor is sensitive to the treatment regime applied. Therefore, two pillars are of importance while developing precision medicine: (1) the individual genome, epigenome, mutations in the cancer and (2) the tumor entity, molecular features and clinical response to cancer therapies (Figure 4). Alongside with DNA sequencing, looking at a patient’s epigenome reinforced the development of precision medicine once again and helped to evaluate new biomarkers. Biomarkers represent a hallmark of precision medicine as they give information on the clinical response to cancer therapies. Recapitulating the development of biomarkers, the term is a portmanteau of “biological marker” that encompasses a wide range of different medical signs. The National Institutes of Health (NIH) biomarkers Definitions Working Group defined a biomarker as “a defined characteristic that is measured as an indicator of normal biological processes, pathogenic processes, or responses to an exposure or intervention, including therapeutic interventions. Molecular, histologic, radiographic, or physiologic characteristics are types of biomarkers, but a biomarker is not an assessment of how an individual feels, functions, or survives.” (Biomarkers Definitions Working Group, 2001).

Nowadays a wide range of biomarkers and their informative value have been defined and a glossary of terms and definitions has been developed by the FDA-NIH Biomarker Working Group called BEST (Biomarkers, Endpoints, and other Tools). A predictive biomarker has been defined as “a biomarker used to identify individuals who are more likely than similar individuals without the biomarker to experience a favorable or unfavorable effect from exposure to a medical product or an environmental agent.” On the other hand, a prognostic biomarker has been described as “a biomarker used to identify likelihood of a clinical event, disease recurrence or progression in patients who have the disease or medical condition of interest” (Cagney et al., 2018). In order to clarify the distinction between those two forms of biomarkers, a prognostic biomarker states distinct disease outcome, whereas predictive biomarkers differentiate between patients who will react or not react to the therapy (Califf, 2018). As described in section “HDACS in Epigenetics and Protein Quality Control Systems” and “Clinical Studies: HDAC Inhibitors in Combination With Modifiers of Protein Quality Control,” epigenetic approaches in cancer treatment and especially HDACi have been implemented and tested in several preclinical and clinical trials. However, open questions on how HDACi function and operate antitumor activity, especially the pathways that are directly linked to HDAC inhibition and tumor cell proliferation, remain to be answered. Due to the paucity of information on HDACi function, it is of importance to detect biomarkers determining the accessibility of tumors during the HDACi treatment regime.

A viable biomarker could detect tumor types that are likely to undergo a favorable clinical response under HDACi therapy. Indeed, the proteasome shuttling factor HR23B seems to play a key role in HDACi-induced apoptosis. In order to identify genes that have an impact on the sensitivity of tumor cells to HDACi, a genome-wide loss-of-function screen was performed. Results revealed not only the role of the UPS in HDACi-induced apoptosis but also the potential of HR23B as a possible biomarker. It has been demonstrated in cells treated with pan-HDACi that HR23B is a sensitivity determinant for HDACi. Therefore, the hypothesis has been proposed that HR23B could function as a biomarker in order to identify tumors that would react favorably to HDACi (Fotheringham et al., 2009). CTCL patients who were treated with vorinostat, showed a positive correlation between HR23B expression levels and therapeutic response. It is therefore likely that HR23B can serve as a predictive biomarker for identifying CTCL patients that respond favorably to HDACi (Khan et al., 2010). The role of HR23B in regulating the biological outcome of treatment with HDACi was then further investigated. Two correlated effects of HR23B in HDACi treated cells were shown: autophagy and apoptosis. While high levels of HR23B cause cells treated with HDACi to undergo apoptosis, low levels of HR23B expression were associated with autophagy (Figure 3). Thus, it was proven that HR23B impacts on the therapy efficacy, as it regulates the switch between apoptosis and autophagy (New et al., 2013). In summary, HR23B represents a promising predictive biomarker and patients with high levels of HR23B would be stratified into a sub-group that would benefit from HDACi therapy, for instance in the PLACARD-trial (NCT03873025) for relapsed or refractory diffuse large B-cell lymphoma (see also section “Novel Strategies of HDAC Inhibitors Affecting Protein Quality Control Pathways”).

Since HDACs have been described to play a role in tumorigenesis, it seems reasonable to measure levels of HDAC enzymes and predict responsive tumor types. It has been demonstrated that specific HDAC isoform expression could be a predictive biomarker. In this study, the influence on knockdown of HDAC1, 2 and 3 isoforms in human cancer cell lines, treated with two unrelated HDACi (belinostat and VPA) have been analyzed. While knockdown of HDAC1 resulted in an increased resistance to belinostat in HeLa cells, no influence was seen in response to either HDAC2 or 3 knockdowns or under VPA treatment. These data suggest that HDAC1 knockdown reduces sensitivity to the HDACi belinostat and in turn high levels of HDAC1 correlate with sensitivity to belinostat treatment (Dejligbjerg et al., 2008). According to these observations, stratification of patients due to their HDAC expression pattern was suggested in colon cancer cell culture models. The specific characterization of class I HDAC isoforms might allow the prediction of individual patient’s prognosis. They observed a negative correlation between HDAC2 expression level and reduced patient survival in patients with CRC (Weichert et al., 2008c). Therefore, they point out how evaluation of HDAC expression profiles would benefit selecting patient populations before HDACi treatment. Similar conclusions were made looking at the class I HDAC expression levels in prostate carcinomas. 192 prostate carcinomas were analyzed using immunohistochemistry and put into subjection to pathological parameters. Again, high expression of HDAC1 and HDAC2 correlated with tumor de-differentiation (Weichert et al., 2008b).

Until now, CTCL represents the malignancy most sensitive to HDACi treatment. Therefore, it is of interest to analyze HDAC profiles in patients suffering from CTCL. In fact, taken 73 CTCL biopsies and analyzing HDAC1, HDAC2, HDAC6, and histone H4 acetylation demonstrated that especially HDAC6 expression correlates with a favorable outcome in CTCL (Marquard et al., 2008). Further studies on HDAC expression levels were performed and implied that depending on the specific tumor entity, different HDAC expression profiles can be observed (Sasaki et al., 2004; Zhang et al., 2004). As the proteome of different tumors can be modified by different HDACs, a comprehensive analysis that could be implemented into clinical routines is desirable. Biomarkers that can be easily detected in peripheral blood mononuclear cells are H3 and H4. They reflect histone acetylation as they are directly modified and regulated by HDACs. Preclinical studies showed a time- and concentration-dependent correlation when histone acetylation is inhibited by HDACi on H3 and H4 (Plumb et al., 2003). However, histone acetylation should only be seen as a surrogate for HDAC inhibition as it does not have the diagnostic value to reflect tumor response. A phase I trial demonstrated the limited validity of H4 measurement. Here, belinostat/PXD101 was used and histone acetylation showed to return to the initial levels within a period of 2 h after drug infusion and displayed to plateau at the maximum tolerated dose (Steele et al., 2008).

As HDACi also intervene in transcriptional regulation, a gene set analysis could give information on HDACi response. Indeed, molecular profiling has been shown to be value in predicting sensitivity during HDACi therapy. In one study, nine genes were tested and identified in NSCLC cell lines under vorinostat and trichostatin A treatment. Three genes were highly associated with drug activity: NAD(P)H quinone dehydrogenase 1 (NQO1), sec homolog A (SEC23A), and proteasome activator subunit 2 (PSME2) (Miyanaga et al., 2008). Further investigations need to be done in order to confirm this nine-gene signature in predicting drug sensitivity. A similar study investigated genes regulated by panobinostat in CTCL patients. In time intervals of 0, 4, 8, and 24 h after panobinostat administration, microarray gene expression profiling was realized. Over time, separate unique gene profiles were reported and 23 genes showed statistical significance. Out of these 23 genes, 4 genes were particularly interesting: guanylate cyclase 1A3 (GUCY1A3), endothelial Tie2/tek ligands angiopoietin-1 (ANGPT1), both associated with angiogenesis and two cell cycle progression genes, transcription factor COUP-TFII (NR2F2) and CCND1 (Ellis et al., 2008). However, a larger study should be executed in order to make a valid statement, thereby also concentrating on cyclin D1 (CCND1), as it is known to be commonly down-regulated by various HDACi (Johnstone, 2002). One challenge to overcome is that due to the wide activity profile of HDACs, gene signatures are likely to vary tremendously depending on tumor type, inhibitor type and concentration. In addition, genes having a prognostic value would be more reasonable to identify than ones that have a response signature.

The future of precision medicine in cancer treatment is highly exciting and promising, although many challenges remain to be solved. Especially biomarkers gain increased attention in order to stratify patients and tumors into sub-groups that are sensitive to HDACi treatment and monitor targeted modulations.

## Conclusion

Over the last decade, we have seen that our scientific knowledge of epigenetics and in particular of HDAC inhibition has been translated into clinical benefit for cancer patients. Epigenetic therapy, such as HDACi, has provided a proof-of-concept of their clinical efficacy. However, one obvious observation made with HDACi and with other epigenetic modifiers such as hypomethylating agents including the DNA methyltransferase inhibitors azacytidine (AZA) or decitabine is that they prove clinical efficacy as single agents in hematological malignancies rather than in solid tumors (Sigalotti et al., 2007). The reasons for this observed discrepancy between hematological malignancies and solid malignancies is still unclear, since many preclinical studies of HDACi alone or in combination with different anti-tumor agents demonstrated their potential to inhibit cell proliferation or even induce apoptosis. One may speculate that the general high cell proliferation in hematological cells foster the effect of epigenetic drugs.

With these observations in mind, combination therapies of HDACi with drugs acting on PQC mechanisms seem to be rational, if cells with a high protein turnover are targeted. A paradigm for this concept is the combination therapy with HDACi and proteasome inhibitors. As outlined in the section “HDAC Inhibitors and Proteasome Inhibitors,” this strategy is proven for MM and is currently tested in various studies for different tumor types and drugs. Our prediction would be that this concept will be successful for those tumor types where a high protein turnover can be observed. This is not only the case in MM with the biogenesis of antibodies, but different cell types excreting proteins such as hepatocytes and cells of the intestine may be more susceptible to drugs acting on PQC systems. Indeed, it has been demonstrated that the half-life of indispensable scaffold proteins in hepatocytes is not necessarily shorter than in highly dividing monocytes (Mathieson et al., 2018). Interestingly, proteins of the UPS are highly abundant in the nucleus. This may reflect the fact that the nucleus is especially vulnerable to imbalances in proteostasis, on the other hand, they may be involved in epigenetic phenomena as well as in DNA damage repair. For instance, the before-mentioned proteasomal shuttling factor HR23B, which plays a role as a potential biomarker in HDACi therapy, is known to mediate DNA damage response (Sugasawa et al., 1998). Usually, transcription factors are rapidly turned over and disturbances in the nuclear UPS will result in differential gene expression, similar to an epigenetic outcome.

As described in sections “HDAC Inhibitors and Bromodomain Inhibitors” and “Novel Strategies of HDAC Inhibitors Affecting Protein Quality Control Pathways,” it has been demonstrated that treatment of cells with clinical concentration of HDACi leads to protein misfolding and aggregation (Olzscha et al., 2017). One could assume that inhibition of molecular chaperones may therefore increase the cytotoxic effects of HDACi. Indeed, some clinical studies provided evidence that this combination therapy may be beneficial for patients with solid tumors, for instance the combination therapy of the HDACi belinostat with the HSP90 inhibitor 17-N-Allylamino-17-demethoxygeldanamycin (17-AAG) (NCT00354185). In order to understand the mechanisms of action, more research is necessary to elucidate the influence of aberrant PTMs on protein folding as well as deciphering which and how molecular chaperones could recognize them.

One of the future challenges will be to identify different tumor types and choose the right therapy for the right patient at the right time. The concept of precision medicine aims not only for stratification of patients; it ensures that each patient benefits from individualized treatment. In order to achieve that, companion diagnostic biomarkers, molecular signatures obtained by genomic and proteomic profiling as well as the health history of the patient should be taken into account (Figure 4). However, a prerequisite for this tailored therapy is a deeper knowledge about the involved pathways of the known HDACs in *H. sapiens* and the involved mechanisms of proteostasis. More basic research is needed to understand how the changing acetylome landscape affects PQC upon HDACi treatment.

## Author Contributions

LK, P-VF, DP, and HO wrote the manuscript. DP created the figures. HO designed the concept and supervised the work.

## Conflict of Interest

The authors declare that the research was conducted in the absence of any commercial or financial relationships that could be construed as a potential conflict of interest.

## Abbreviations

ABL: Abelson leukemia
AD: Alzheimer’s disease
AKT: AKT Serine/Threonine Kinase
ALS: amyotrophic lateral sclerosis
AML: acute myeloid leukemia
ANGPT1: endothelial Tie2/tek ligands angiopoietin-1
Apaf-1: apoptotic protease activating factor 1
ATAT1: alpha-tubulin N-acetyltransferase
ATF6: activating transcription factor 6
ATG: autophagy-related genes
ATP: adenosine triphosphate
BBB: blood-brain barrier
Bcl-2: B-cell lymphoma 2
BCR-ABL: breakpoint cluster region protein Abelson murine leukemia viral oncogene homolog
BET: bromodomain and extra-terminal domain
BiP: binding immunoglobulin protein
BNIP3: Bcl-2/adenovirus E1B 19 kDa protein-interacting protein 3
BCR: breakpoint cluster region
BRAF: proto-oncogene B-Raf
BRD: bromodomain
cAMP: cyclic adenosine monophosphate
CBP: CREB binding protein
CCND1: cyclin D1
CDK: cyclin-dependent kinase
CHIP: carboxy terminus of heat shock protein 70-interacting protein
CMA: chaperone-mediated autophagy
c-Met: tyrosine-protein kinase Met
CRC: colorectal cancer
CREB: cAMP response element-binding protein
CRL: cullin-ring E3 ligases
CTCL: cutaneous T cell lymphoma
DNA: deoxyribonucleic acid
ER: endoplasmic reticulum
ERB-B2: Erb-B2 receptor tyrosine kinase 2
ETO: eight-twenty-one
FDA: U.S Food and Drug Administration
FLT3: FMS like tyrosine kinase 3
GBM: glioblastoma
GRP78: Glucose-regulated protein 78
GUCY1A3: guanylate cyclase 1A3
H3: histone H3
H4: histone H4
HCQ: hydroxychloroquine
HDA1: histone deacetylase 1
HDAC: histone deacetylase
HDACi: histone deacetylase inhibitor
HL: Hodgkin lymphoma
HR: homologous recombination
HR23B: nucleotide excision repair protein homolog B
HSP: heat shock protein
HSP70: heat shock protein 70
HSP90: heat shock protein 90 hypoxia-inducible factor (HIF)-1 α
IRE1: inositol-requiring enzyme 1
IRF4: interferon regulatory factor 4
Ki: inhibitory constant
MDM2: mouse double minute 2
MM: multiple myeloma
mTOR: mammalian target of rapamycin
MYC: myelocytomatosis
NADPH: nicotinamide adenine dinucleotide phosphate
NAE: NEDD8-activating enzymes
NEDD8: neural-precursor-cell-expressed developmentally down-regulated 8
NF- κ B: nuclear factor “kappa-light-chain-enhancer” of activated B-cells
NHEJ: non-homologous end-joining recombination
NIH: National Institutes of Health
Nox4: NADPH oxidase 4
NQO1: NAD(P)H quinone dehydrogenase 1
NR2F2: nuclear receptor subfamily 2 group F member 2
NSCLC: non-small-cell lung cancer
NUT: nuclear protein in testis
PERK: the protein kinase RNA-like endoplasmic reticulum kinase
PFS: progression-free survival
PI: proteasome inhibitor
PQC: protein quality control
PSME2: proteasome activator subunit 2
PTCL: peripheral T-cell lymphoma
PTM: posttranslational modification
RING: really interesting new gene
SAHA: suberoylanilide hydroxamic acid
SEC23A: sec homolog A
COPII: coat complex component
sHSP: small heat shock protein
SOD1: superoxide dismutase 1
SP1: specific protein 2
SUMO: small ubiquitin like modifier
UPR: unfolded protein response
UPS: ubiquitin proteasome system
TSA: trichostatin A
VEGFR: vascular endothelial growth factor receptor
VPA: valproic acid.
